# The Association Between Maternal Subclinical Hypothyroidism and Growth, Development, and Childhood Intelligence: A Meta-analysis

**DOI:** 10.4274/jcrpe.4931

**Published:** 2018-05-18

**Authors:** Yahong Liu, Hui Chen, Chen Jing, FuPin Li

**Affiliations:** 1 The Second Hospital of Lanzhou University, Department of Pediatrics, Lanzhou, Gansu, China; 2 The Second Hospital of Lanzhou University, Department of Endocrinology, Lanzhou, Gansu, China; 3 Nanfang College of Sun Yat-sen University Faculty of Health and Nursing, Guangzhou, Guangdong, China; 4 Gansu Provincial Maternity and Childcare Hospital, Lanzhou, Gansu, China

**Keywords:** Gestation, subclinical hypothyroidism, child development, meta-analysis

## Abstract

**Objective:**

To explore the association between maternal subclinical hypothyroidism (SCH) in pregnancy and the somatic and intellectual development of their offspring.

**Methods:**

Using RevMan 5.3 software, a meta-analysis of cohort studies published from inception to May 2017, focusing on the association between maternal SCH in pregnancy and childhood growth, development and intelligence, was performed. Sources included the Cochrane Library, Pub-Med, Web of Science, China National Knowledge Infrastructure and Wan Fang Data.

**Results:**

Analysis of a total of 15 cohort studies involving 1.896 pregnant women with SCH revealed that SCH in pregnancy was significantly associated with the intelligence (p=0.0007) and motor development (p<0.00001) of the offspring. SCH was also significantly associated with the child’s weight in four studies involving 222 women (p=0.02). Maternal SCH in pregnancy was identified as a risk factor for fetal growth restriction with a combined relative risk (RR) value of 2.4 [95% confidence interval (CI): 1.56, 3.7]. Meta-analysis of 10 studies that provided numbers of preterm infants revealed a significant association between maternal SCH in pregnancy and premature delivery, with a combined RR of 1.96 (95% CI: 1.34, 2.88). There was a significant effect of maternal SCH in pregnancy on fetal distress *in utero* (p=0.003).

**Conclusion:**

Maternal SCH in pregnancy is associated with increased risk of adverse neonatal outcomes, including delayed intellectual and motor development, low birth weight, premature delivery, fetal distress and fetal growth restriction.

## What is already known on this topic?

Thyroid hormone plays an important role in the differentiation, development and maturation of tissues. Insufficiency or lack of thyroid hormones during pregnancy have a negative effect on fetal neurological and intellectual development. Many studies suggest that hypothyroidism in pregnancy can increase the risk of adverse outcomes of pregnancy. To this date, there is no established consensus on routine screening for subclinical hypothyroidism in pregnant women due to lack of credible evidence.

## What this study adds?

Our meta-analysis showed that maternal subclinical hypothyroidism in pregnancy is associated with an increased risk of several adverse neonatal outcomes. Thus, it is necessary to assess thyroid function in pregnant women and intervene when necessary. Because of the high methodological quality of the included trials, our data provide supportive evidence for initiating further investigations.

## Introduction

Thyroid hormone (TH) promotes growth via its effect on protein synthesis. It also plays an important role in differentiation, development and tissue maturation (1). TH is essential for brain cell proliferation. Prior to gestational week 20, TH-dependent brain development fully or partly depends on maternal TH (2,3). Many studies provide evidence that hypothyroidism in pregnancy may increase the risk of adverse outcomes, including premature delivery, low birth weight (LBW), fetal demise and disrupted neurological/intellectual development of the fetus (4,5,6). Subclinical hypothyroidism (SCH) is defined as an elevated serum thyroid stimulating hormone (TSH) level in the context of normal triiodothyronine (T_3_) and tetraiodothyronine (T_4_) levels (7,8,9). Presence of SCH in pregnancy can be expected to have adverse effects on the growth and development of the fetus. In one survey, there was a higher incidence of maternal SCH in pregnancy (11.3%) compared with clinical hypothyroidism in pregnancy (2.4%) (10). Intelligence quotient scores in the offspring of women with untreated SCH were lower than those of the children of normal pregnant women (11). Currently, there is no consensus regarding routine SCH screening in pregnant women, owing to lack of credible evidence. Although many studies have investigated the effects of maternal SCH on childhood growth, development and intelligence level, the results have not been uniform. We therefore thought it would be worthwhile to conduct this meta-analysis of published studies to provide evidence-based research on the effects of maternal SCH on the growth, development and intelligence of the offspring.

## Methods

This meta-analysis was based on the reports of domestic and international cohort studies that evaluated the effects of maternal SCH in pregnancy on childhood growth, development and intelligence. Only peer-reviewed articles published in English and Chinese were included in the analysis. The analysis only included studies on women with SCH in pregnancy with singleton fetuses. All subjects, apart from having SCH, were otherwise healthy. Maternal SCH was defined as having a serum TSH level higher than the upper limit of pregnancy-specified reference value (the 97.5^th^ percentile of normal) and a serum free thyroxine (FT_4_) level within normal range (between 2.5^th^ to 97.5^th^ percentiles of normal value) (12) or a TSH level between 2.5 and 10 mIU/L and a normal FT_4_ level according to the definition established by the American Thyroid Association (13). All analyses were based on previous published studies, thus no ethical approval and patient consent are required.

We evaluated the following outcomes: growth; development and intelligence levels of the child (including growth, cognition and intelligence) measured by intelligence tests such as the Bayley Scales or the Gesell Scales. The Bayley Scales of Infant Development is one of the most psychometrically valid measurements for examining mental and psychomotor development in 1 to 30 months-old infants. This test consists of a mental scale (163 items) and a psychomotor scale (81 items) used to assess cognitive development, including visual performance function, memory, first verbal outcomes and fine and gross motor development. Age at assessment-adjusted raw scores were centered to a mean of 100 with a standard deviation of 15 to calculate index scores (mental and psychomotor scores). Gesell Scales can reflect the development level of children from five aspects: gross motor quotient; fine motor quotient; adaptive behavior quotient; individual social behavior quotient; and language quotient. Birth weight (BW); LBW (≤2500 g); premature birth (<37 weeks gestation); intrauterine distress (variously defined); and fetal growth restriction (IUGR, variously defined) were also considered in the analysis.

The following studies were excluded: studies which included pregnant women treated with thyroxine; studies that did not include pregnant women with normal thyroid function as controls; non-cohort studies; publications containing duplicate data from the same study; studies for which the full text was not available; studies published only as abstracts; studies that did not provide initial data and did not respond to our requests for more information; and studies with inconsistent data.

Cochrane Library, Pub-Med, Web of Science, China National Knowledge Infrastructure and Wan Fang Data were searched from the inception of each database to May 2017 for relevant studies. Search words in Chinese included: pregnancy, gestation, SCH, growth and development, intelligence; search words in English included pregnancy, pregnancies, gestation, SCH, cognition, growth, intelligence, premature birth, premature delivery, premature labor, preterm delivery, preterm birth, preterm labor, fetal distress, intrauterine distress, fetal growth restriction. As an example, the specific search strategy for Pub-Med is shown in Table 1.

Two investigators worked independently to screen articles according to the inclusion and exclusion criteria. Disagreements were resolved by discussion with a third reviewer, also an expert in the field. Extracted data included authors, year of publication, sample size, gestational age at screening, intelligence score, BW, preterm delivery, intrauterine distress and fetal growth restriction.

The quality of selected studies was assessed independently by two investigators, using the Newcastle-Ottawa Scale (NOS) for cohort studies. The highest NOS score was 9 points, and consisted of the following three aspects: the definition and selection of subjects in the case and control groups (0-4 points); the comparability between study groups (0-2 points); and determination of exposure factors (0-3 points). A paper that scored ≥7 points or more was considered “high-quality”.

### Statistical Analysis

Meta-analysis was performed with Rev Man software (edition 5.3). Qualitative and quantitative analyses were performed, and 95% confidence intervals (CI) (95% CI) were calculated. Heterogeneity analysis was performed with the I^2^ test. Meta-analysis was performed with a fixed effect model for studies without heterogeneity (I^2 ^<50%), and a random effect model for studies with heterogeneity (I^2^ ≥50%) after data combination (14). Descriptive analysis only was conducted for clinical trials with data not suitable for meta-analysis. Effect size was represented as relative risk (RR) for categorical variables and as standard mean difference for continuous variables, with 95% CI.

## Results

**Figure 1:**
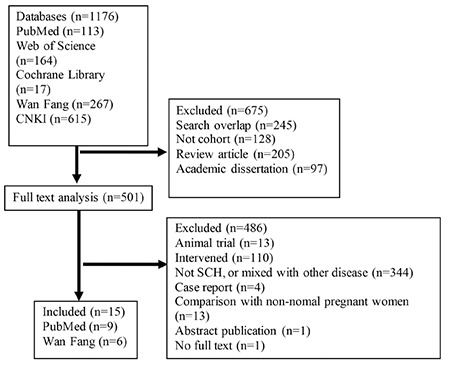
Flow chart of the article selection process CNKI: China National Knowledge Infrastructure, SCH: subclinical hypothyroidism

A total of 1.176 articles were identified with the prespecified search strategy. Ultimately, 15 articles were included in the analysis after step-by-step screening (5,10,15,16,17,18,19,20,21,22,23,24,25,26,27). The screening flow chart is shown in Figure 1.

**Basic characteristics and quality evaluation of included studies: **Of the 15 studies included, nine (5,10,15,16,17,18,19,20,21) were published in English and the remaining six were published in Chinese. A total of 1.896 patients met the inclusion criteria and were included in the study, with 37.968 controls. Fetal intrauterine distress was reported in five studies (17,19,20,22,27). The number of premature infants in various groups was reported in 10 studies (5,10,15,16,19,20,21,22,26,27). Neonatal BW was reported in four studies (10,19,24,26). The number of LBW neonates was reported in seven studies (5,15,17,20,21,22,27). Childhood intelligence was measured at age 12 to 30 months in three studies (18,24,25). In this meta-analysis, we chose cohort studies with high methodological quality. The principal characteristics and NOS scores are displayed in Table 2 and 3.

**Maternal SCH in pregnancy and child intellectual and motor development:** Because child intelligence and motor ability develop as children mature, studies with children of similar age were included in this meta-analysis (Table 4). In these three studies, the age range was 12 to 30 months. Intellectual and motor development were measured by Bayley Scales. The intellectual and motor development level of the children were compared between the SCH group and the control group. A fixed effect model was used because of the heterogeneity among studies (p=0.17, I^2=^47%). There was a significant association between maternal SCH in pregnancy and child intellectual development (MD=-6.08, 95% CI: -9.57~-2.58, p=0.0007) and child motor development (MD=-7.29, 95% CI: -10.30~-4.28, p<0.00001).

**Figure 2:**
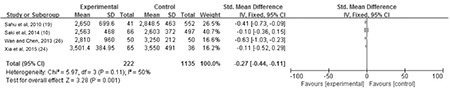
The forest plot of relative risk and 95% confidence interval of pooled studies comparing pregnant women with subclinical hypothyroidism to euthyroid pregnant women for risk of birth weight SD: standard deviation, CI: confidence interval

**The association between maternal SCH and BW:** Data on BW were provided in four studies. The meta-analysis of these four studies revealed moderate heterogeneity (p=0.11, I^2=^50%). Thus, a fixed effect model was used. We found a significant effect of maternal SCH in pregnancy on BW (MD=-0.27, 95% CI: -0.44~-0.11, p=0.001) (Figure 2).

**The association between maternal SCH and LBW:** Data on LBW were provided in seven studies. We found moderate heterogeneity among them (I^2=^58%). Thus, a random-effect model was used. Maternal SCH was a risk factor for LBW (combined RR: 1.78, 95% CI: 1.04-3.07, p=0.04). Figure 3A shows the association between maternal SCH and LBW. We also made a funnel plot as shown in Figure 3B. The funnel plot of this study is asymmetrical, indicating that there may be a publication bias.

**Figure 4:**
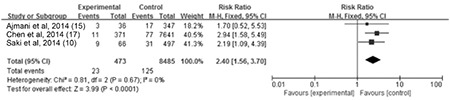
The forest plot of relative risk and 95% confidence of pooled studies comparing pregnant women with subclinical hypothyroidism to euthyroid pregnant women for risk of fetal growth restriction CI: confidence interval

**The association between maternal SCH and fetal growth restriction:** Data on fetal growth restriction was provided in three studies. The meta-analysis of these studies demonstrated a significant correlation between maternal SCH and fetal growth restriction, with no heterogeneity among results (I^2=^0%). Thus, a fixed-effect model was used and determined a combined RR of 2.4 (95% CI (1.56, 3.7), p<0.0001), as shown in Figure 4.

**Figure 5:**
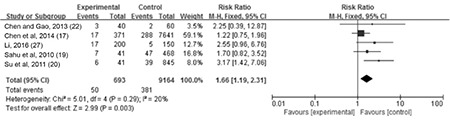
The forest plot of relative risk and 95% confidence interval of pooled studies comparing pregnant women with subclinical hypothyroidism to euthyroid pregnant women for risk of fetal intrauterine distress CI: confidence interval

**The association between maternal SCH and fetal intrauterine distress: **Data on fetal distress were provided in five studies. Meta-analysis of these studies showed minor heterogeneity among results (I^2^=20%). Thus, a fixed-effect model was used and demonstrated a combined RR of 1.66 [95% CI (1.19, 2.31), p=0.003], as shown in Figure 5.

**The association between maternal SCH and premature delivery: **Data on the number of preterm and term infants were provided in ten studies. Meta-analysis of these studies revealed moderate heterogeneity (p=0.006, I^2^=61%). Thus, a random-effect model was used and demonstrated a combined RR of 1.96 [95% CI (1.34, 2.88), p=0.0006]. The forest plot and funnel forest plot are displayed in Figure 6A and 6B. The funnel plot of this study is symmetrical, indicating that there is no publication bias.

## Discussion

TH is an important hormone in human metabolism throughout the whole lifespan. It is a particularly important factor for brain development and corporal growth in the fetus. The first stage of the fetal brain growth-spurt occurs during the first and second trimesters of pregnancy. At this time TH is provided to the fetus mainly from transplacental delivery of THs (mainly T_4_), because fetal thyroid follicular epithelial cells are immature and cannot yet synthesize TH during the first 12 weeks of pregnancy (3). An insufficient supply of maternal THs during this period can cause significant and irreversible neurodevelopmental defects. Vulsma et al (28) showed that the umbilical cord level of T_4_ in fetuses with congenital hypothyroidism due to a total organification defect or thyroid agenesis was 30% to 60% that of normal fetuses, suggesting that maternal input of T_4_ continues until birth. Therefore, brain development during late pregnancy is driven by both fetal and maternal THs. Deficient maternal THs in late pregnancy can cause neurodevelopmental defects, although the effect may not be as serious as the impact of maternal thyroid deficiency during the first trimester of pregnancy. Children of pregnant women with overt hypothyroidism were found to have a lower level of physical and intellectual development, as well as a lower level of responsiveness to external stimuli compared with children of pregnant women with normal thyroid function (4,5,6). It is unclear whether the same effect is true for children of women with SCH. Some investigators speculate that although women with SCH may have normal FT_4_ levels, the increased TSH suggests these women need higher TH levels to ensure fetal development (29). Currently, overt hypothyroidism in pregnancy is an indication for TH replacement therapy. For women with SCH, however, it remains unclear whether TH supplementation would improve the developmental status of the child. Some investigators believe that thyroxine treatment would ameliorate adverse pregnancy outcomes, and other investigators believe the contrary (30,31). Our meta-analysis suggests that SCH is associated with delayed child intellectual and motor development, but the follow-up time of studies included in this meta-analysis was only up to two years of age, thus the impact of maternal SCH in the long-term was not investigated.

T_3_ and T_4_ promote the growth of long bones and teeth by stimulating the development of ossification centers. In addition, THs enhance glycogenolysis and inhibit glycogen synthesis by enhancing intestinal glucose absorption as well as by increasing glucose use by peripheral tissues. Thus, TH deficiency is a risk factor for fetal growth restriction and for LBW, both of which are risk factors for unsatisfactory neurological, motor, and intellectual development (2,32,33). Ohashi et al (34) reported a probability of 25% for fetal growth restriction in pregnant women with abnormal thyroid function and a probability of 16.25% for fetal growth restriction in pregnant women with SCH. Our study showed a 2.4-fold risk increase for fetal growth restriction in pregnant women with SCH as compared to pregnant women with normal thyroid function and a 1.78-fold risk for LBW. These results are consistent with those reported by Leung et al (35).

Zhang (36) reported a 6.62 - 7.86% incidence of abnormal thyroid function, SCH being the most common abnormality, prior to gestational week 20 in women without a previous history or family history of thyroid disease. SCH in pregnancy may be asymptomatic but, nevertheless, can negatively affect fetal neurodevelopment. Therefore, screening for thyroid abnormalities should be performed in the first trimester of pregnancy and if the TSH shows an abnormal level, more attention should be paid to the growth and development of the fetus. Some authors suggest that screening for SCH in pregnancy may be a cost-effective strategy in a wide range of circumstances (37). The effect of the intervention depends on the timing during gestation. Therefore, we suggest that there is an urgent need for large-scale, randomized trials to measure the intelligence of children whose mothers with SCH who were treated with thyroxine during pregnancy versus normal children,in theory,it should also be compared with non-intervention group, but this does not sound ethical to us, we may only be able to verify it in animal experiments.

Some studies suggest that SCH or elevated TSH in pregnancy result in premature delivery (15,38,39) while other studies reached contrary conclusions (10,19). Our meta-analysis showed that maternal SCH is associated with premature delivery. Maraka et al (14) also published a meta-analysis, showing that SCH during pregnancy is associated with multiple adverse maternal and neonatal outcomes. The effect of levothyroxine therapy in preventing these adverse outcomes remains uncertain. It is therefore necessary to do a large-scale trial to assess the value of levothyroxine therapy. In this study different TSH strata should be explored to identify the optimal treatment threshold, where the benefits of levothyroxine use outweigh the risks (40).

### Study Limitations

The selection bias was small in this study due to a review of a vast literature and strict adherence to pre-specified inclusion and exclusion criteria. However, every meta-analysis has limitations. In our study, we did not take into account the effects of anti-thyroid antibodies, including anti-thyroglobulin antibodies and anti-thyroid peroxidase antibodies. As a result, we might have underestimated the effect of maternal SCH on growth and development or intelligence level. In addition, the studies included in our analysis did not provide data on family economic status, parental education level, or the environment. All of these may be confounding factors affecting child development. Also, measurement methods and measuring instruments varied across the studies included in our analysis. This, too, might have affected the results.

Our meta-analysis suggests that maternal SCH is associated with fetal growth restriction, impaired intellectual and motor development, LBW, premature delivery and fetal distress. Therefore, more extensive screening for thyroid function during or even prior to pregnancy may be especially important for improved outcomes. We speculate that TH supplementation may promote normal fetal development and may prevent adverse pregnancy outcomes. As this is only speculation, large-scale randomized trials are needed.

## Conclusion

Our meta-analysis showed that maternal SCH in pregnancy is associated with an increased risk of several adverse neonatal outcomes. Thus, it is necessary to assess thyroid function in pregnant women and intervene when necessary. Because of the high methodological quality of the included trials, our data provide supportive evidence for initiating further investigation. Additional cohort studies including large numbers of participants are needed to guide future investigation.

## Figures and Tables

**Table 1:**
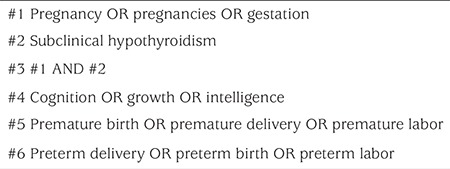
PubMed search strategy

**Table 2:**
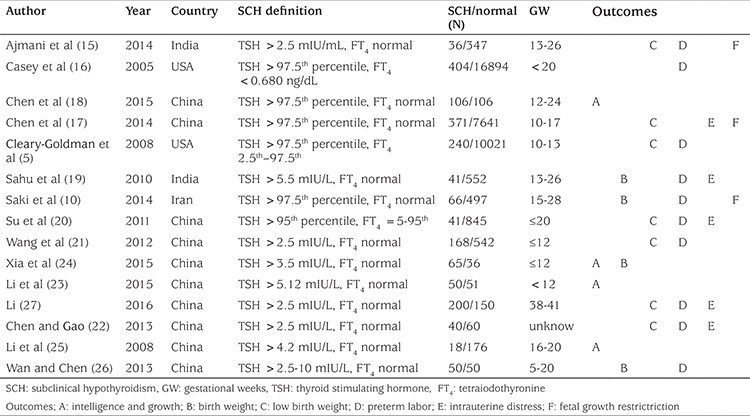
Study characteristics

**Table 3:**
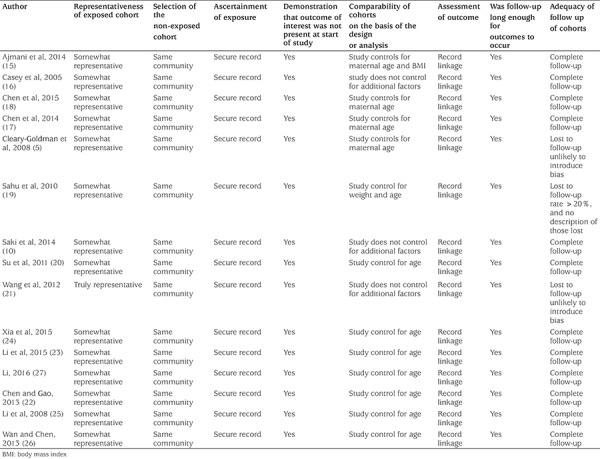
Risk of bias

**Table 4:**
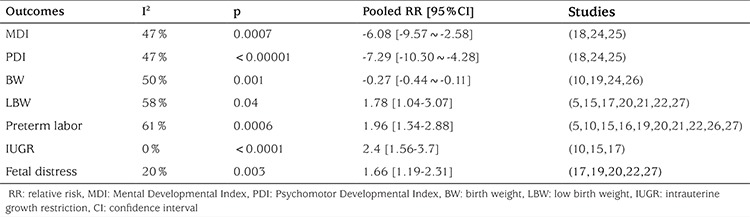
Pooled relative risk with 95% confidence interval comparing pregnant women with subclinical hypothyroidism to pregnant euthyroid women

**Figure 3A:**
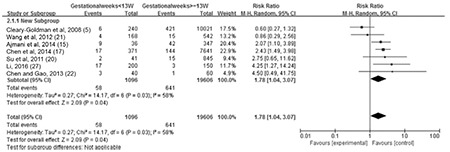
The forest plot of relative risk and 95% confidence interval of pooled studies comparing pregnant women with subclinical hypothyroidism to euthyroid pregnant women for risk of low birth weight CI: confidence interval

**Figure 3B:**
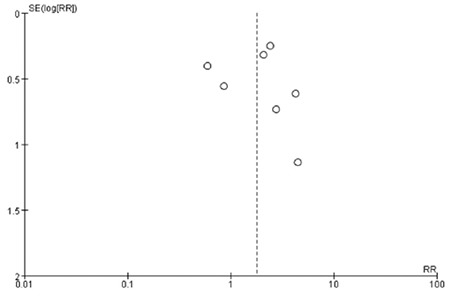
The funnel plot of pooled studies comparing pregnant women with subclinical hypothyroidism to euthyroid pregnant women for risk of low birth weight RR: relative risk

**Figure 6A:**
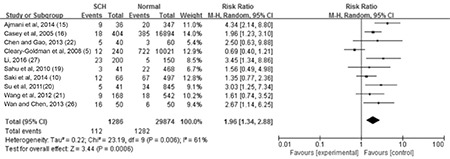
The forest plot of relative risk and 95% confidence interval of pooled studies comparing pregnant women with subclinical hypothyroidism to euthyroid pregnant women for risk of premature delivery CI: confidence interval

**Figure 6B:**
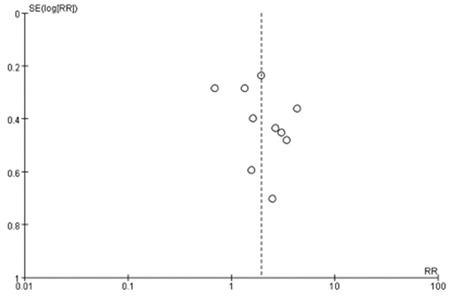
The funnel plot of pooled studies comparing pregnant women with subclinical hypothyroidism to euthyroid pregnant women for risk of premature delivery RR: relative risk
